# Haemorrhagic cholecystitis treated with endovascular embolization

**DOI:** 10.1093/jscr/rjad377

**Published:** 2023-07-11

**Authors:** Joseph Morrow, Christopher Garvey, J Mark Ryan

**Affiliations:** Department of Interventional Radiology, St. James’s Hospital, Dublin 8, Leinster, Ireland; Department of Interventional Radiology, St. James’s Hospital, Dublin 8, Leinster, Ireland; Department of Interventional Radiology, St. James’s Hospital, Dublin 8, Leinster, Ireland

## Abstract

Haemorrhagic cholecystitis is a rare complication of acute cholecystitis. Traditionally, treatment has been with emergency cholecystectomy. Endovascular management of haemorrhage allows the patient to be optimized for surgery at a later date. Our case presents a 52-year-old woman with haemorrhagic cholecystitis who underwent endovascular coil embolization of the cystic artery in interventional radiology. Further complications later ensued including a haematoma in the gallbladder fossa and a bile leak into the peritoneal cavity. As a result, the patient had an endoscopic retrograde cholangiopancreatography (ERCP) with placement of a covered stent into the extrahepatic bile ducts. The patient later developed abscess formation in the gallbladder fossa, which was managed with a percutaneous pigtail drain. Following clinical and radiological improvement, the patient was discharged with the gallbladder fossa drain and biliary stent *in situ* to await elective cholecystectomy. Endovascular embolization is a useful alternative, in the acute setting, to emergency surgical cholecystectomy.

## INTRODUCTION

Haemorrhagic cholecystitis is a rare complication of acute cholecystitis. Traditionally, treatment has been with emergency cholecystectomy [1]. Endovascular management of haemorrhage allows the patient to be optimized for surgery at a later date.

We present a case of haemorrhagic cholecystitis initially treated with endovascular embolization.

## CASE REPORT

A 52-year-old woman was referred to our third level institution from a smaller hospital in our group. Two months prior, the patient underwent coronary artery stenting and was commenced on dual antiplatelet therapy. One month prior, she underwent ultrasound liver due to deranged liver function tests. This demonstrated cholelithiasis without evidence of acute cholecystitis.

**Figure 1:**
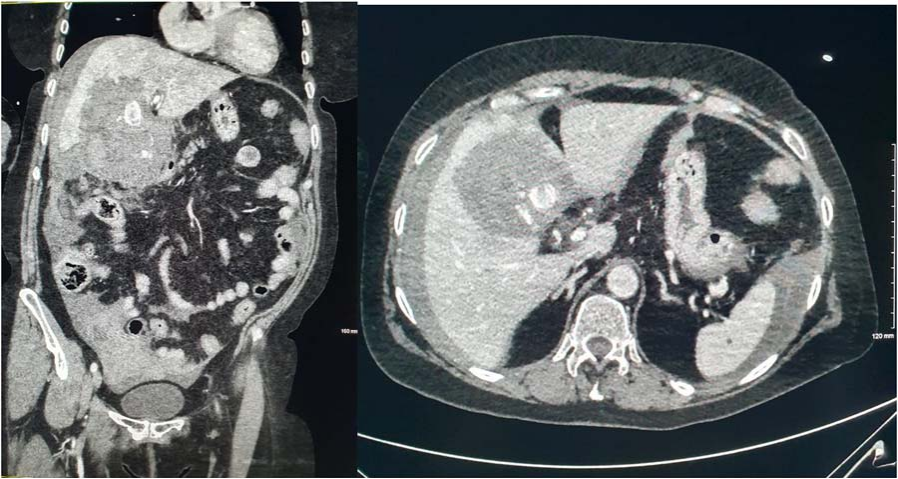
CT abdomen and pelvis with intravenous contrast in the portal venous phase was performed demonstrating perforated acute calculous cholecystitis and active haemorrhage.

She presented on this occasion with right upper quadrant pain and collapse. Computed tomography (CT) abdomen and pelvis with intravenous contrast in the portal venous phase was performed, demonstrating perforated acute calculous cholecystitis and active haemorrhage (Fig. 1). At this point, Interventional Radiology and Upper gastrointestinal (GI) Surgery in our institution were contacted, and the patient was transferred for emergency embolization. The patient was haemodynamically unstable, so the massive transfusion protocol was commenced.

**Figure 2:**
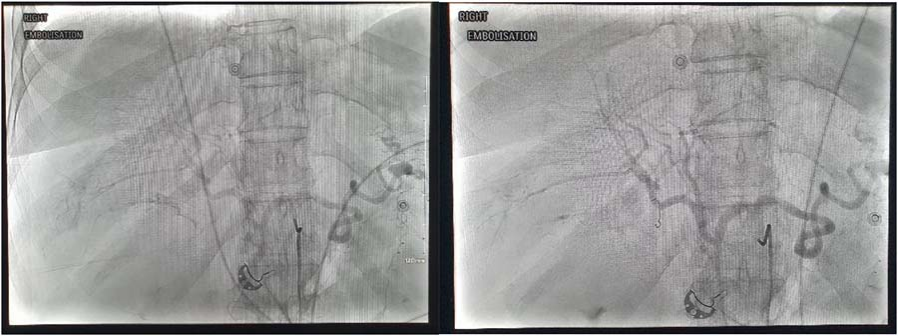
DSA images demonstrating active haemorrhage from the cystic artery and subsequent coil embolization with cessation haemorrhage.

Right common femoral artery access was achieved, followed by Mickelson catheter access to celiac axis. Digital subtraction angiography (DSA) demonstrated active haemorrhage arising from the cystic artery (Fig. 2). A Renegade HiFlow microcatheter (Boston Scientific) was advanced into the cystic artery arising from the right hepatic artery. A repeat DSA in this position demonstrated multiple points of active haemorrhage around the gallbladder with two main branches arising from a central trunk. A single 3 × 3.3 mm Vortx coil (Boston Scientific) was deployed into the proximal cystic artery. A repeat DSA through the microcatheter in the right hepatic artery demonstrated the cessation of flow in the cystic artery and no further active haemorrhage (Fig. 2).

Plans to perform a cholecystectomy were deferred when the gallbladder lumen could not be differentiated from pericholecystic haematoma on ultrasound. The patient quickly stabilized post embolization and returned to the ward.

**Figure 3:**
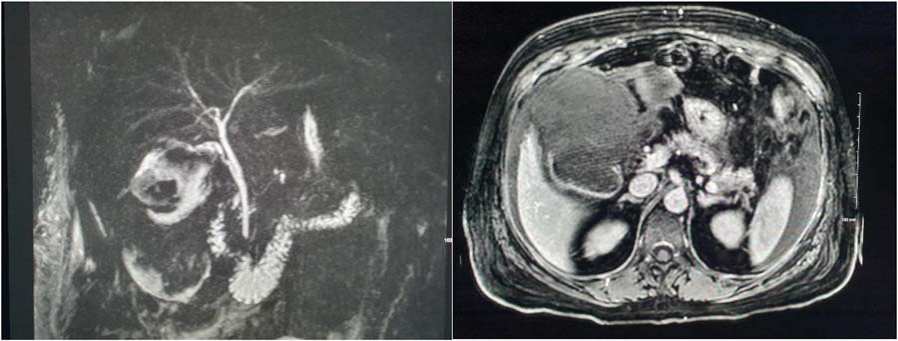
MRCP and post contrast MRI images demonstrating the wide mouthed frank disruption of gallbladder wall.

**Figure 4:**
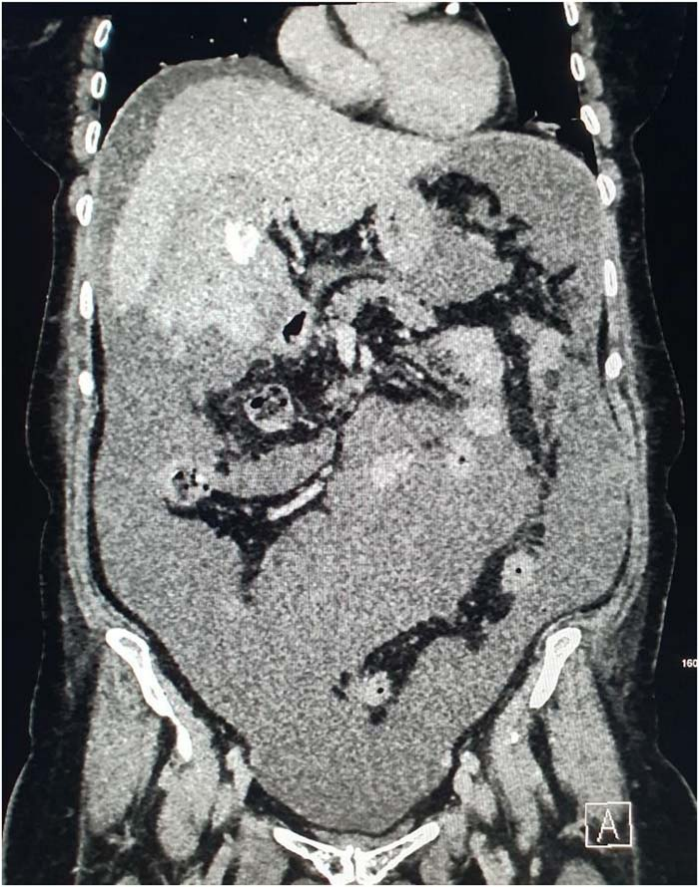
CT demonstrating large volume of bilious peritoneal fluid with haematoma and calculus in the gallbladder fossa.

**Figure 5:**
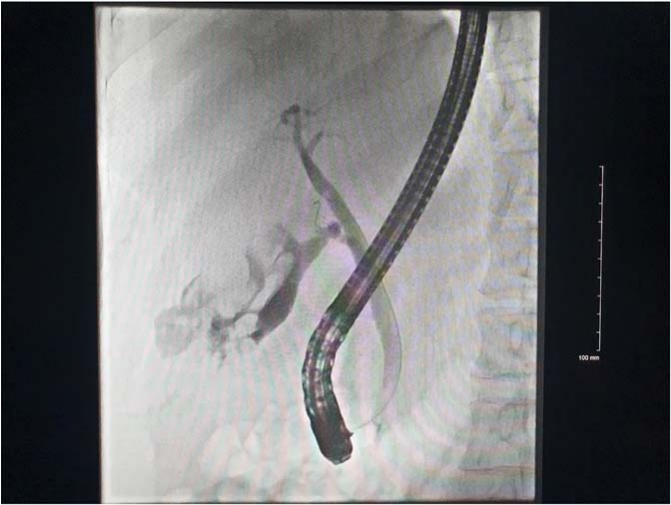
Fluoroscopic ERCP image demonstrating gallbladder perforation.

Magnetic resonance imaging (MRI) of the liver and magnetic resonance cholangiopancreatography (MRCP) were performed in the following days, which demonstrated a frank rupture of the gallbladder and a patent biliary tree (Fig. 3). After a few more days, the patient became peritonitic and a further CT was performed. This demonstrated a moderate volume of peritoneal free fluid and haematoma in the gallbladder fossa (Fig. 4). Ultrasound-guided drainage of peritoneal fluid was performed producing bilious fluid. ERCP was performed, which demonstrated ongoing leak from the gallbladder fossa (Fig. 5) and allowed placement of a covered biliary stent to divert flow away from the cystic duct.

**Figure 6:**
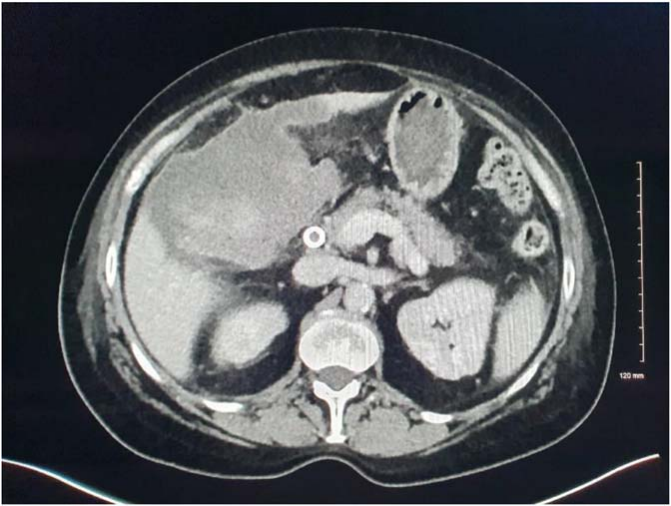
CT demonstrating superinfection of the gallbladder fossa haematoma and abscess formation.

**Figure 7:**
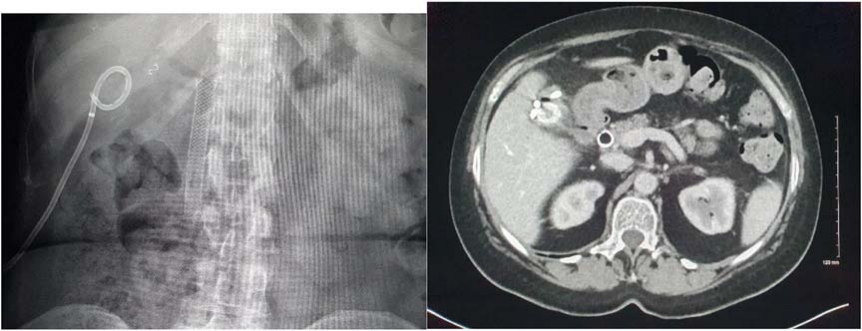
Radiographic and CT images demonstrating the patients’ hardware at discharge with an endoscopic biliary stent and gallbladder fossa pigtail drain *in situ*.

CT of the abdomen was performed the following week due to pyrexia and ongoing pain. This demonstrated abscess formation at the site of haematoma in the gallbladder fossa (Fig. 6). A locking pigtail drain was placed in the gallbladder fossa collection under ultrasound guidance. Slow drainage of bloodstained bilious fluid from the right upper quadrant with resolution was demonstrated on CT the following month (Fig. 7). The patient was discharged with gallbladder (GB) fossa drain and biliary stent *in situ* to await elective cholecystectomy.

## DISCUSSION

Haemorrhagic cholecystitis is a rare complication of acute cholecystitis with reported mortality rate of 15–20%. This case demonstrates the potential value of initial treatment with endovascular embolization. Haemorrhage can occur into the peritoneum where large volumes can result in hypovolemic shock and haematoma formation. Haemorrhage can also occur into the hepatic parenchyma and biliary system. Haemorrhage into the biliary system can result in biliary obstruction and signs of upper GI haemorrhage. Multiphasic CT is the imaging method of choice. It can demonstrate the site of haemorrhage and aid the planning of embolization or surgery. Direct angiography would only be performed as part of a therapeutic procedure.

The cystic artery arises from the right hepatic artery in 79% and it is worth remembering that multiple cystic arteries are found in 8.9% of patients [2]. Haemorrhagic cholecystitis can occur due to calculi as in this case but also due to direct trauma, tumour or hypocoagulable states [3]. It is difficult to estimate the rate of haemorrhagic cholecystitis or morbidity and mortality due to the literature consisting largely of isolated case reports [3]. Endovascular embolization is a useful alternative, in the acute setting, to emergency surgical cholecystectomy.

## CONFLICT OF INTEREST STATEMENT

None declared.

## FUNDING

None.
